# Supplementary material for: HIVprotI: an integrated web based platform for prediction and design of HIV proteins inhibitors
Source: J Cheminform. 2018 Mar 9;10:12. doi: 10.1186/s13321-018-0266-y (PMC5845081; doi:10.1186/s13321-018-0266-y)
Supplement: Supplementary file 2 — Additional file 2. Source code of HIVProtI web server. [file 13321_2018_266_MOESM2_ESM.zip › HIVprotI_Source-code/hivprotI/weka-3-6-6/doc/weka/gui/beans/AbstractTrainAndTestSetProducer.html]

AbstractTrainAndTestSetProducer


---


|  |  |  |
| --- | --- | --- |
| **Overview** \| **Package** \| **Class** \| **Tree** \| **Deprecated** \| **Index** \| **Help** \| **Weka's home** | |  |
| **PREV CLASS**   **NEXT CLASS** | **FRAMES**    **NO FRAMES**     **All Classes** |  |
| SUMMARY: NESTED \| FIELD \| CONSTR \| METHOD | DETAIL: FIELD \| CONSTR \| METHOD |  |


---


## weka.gui.beans Class AbstractTrainAndTestSetProducer

```
java.lang.Object
  java.awt.Component
      java.awt.Container
          javax.swing.JComponent
              javax.swing.JPanel
                  weka.gui.beans.AbstractTrainAndTestSetProducer
```

**All Implemented Interfaces:**: java.awt.image.ImageObserver, java.awt.MenuContainer, java.io.Serializable, java.util.EventListener, javax.accessibility.Accessible, BeanCommon, DataSourceListener, TestSetProducer, TrainingSetProducer, Visible

**Direct Known Subclasses:**: CrossValidationFoldMaker, TrainTestSplitMaker

---

``` public abstract class AbstractTrainAndTestSetProducer extends javax.swing.JPanel implements Visible, TrainingSetProducer, TestSetProducer, BeanCommon, java.io.Serializable, DataSourceListener ```

Abstract base class for TrainAndTestSetProducers that contains default
implementations of add/remove listener methods and defualt
visual representation.

**Version:**
:   $Revision: 1.5 $

**Author:**
:   Mark Hall

**See Also:**: Serialized Form

---

**Nested Class Summary**

**Nested classes/interfaces inherited from class javax.swing.JComponent**

`javax.swing.JComponent.AccessibleJComponent`

**Nested classes/interfaces inherited from class java.awt.Component**

`java.awt.Component.BaselineResizeBehavior`


**Field Summary**

**Fields inherited from class javax.swing.JComponent**

`TOOL_TIP_TEXT_KEY, UNDEFINED_CONDITION, WHEN_ANCESTOR_OF_FOCUSED_COMPONENT, WHEN_FOCUSED, WHEN_IN_FOCUSED_WINDOW`

**Fields inherited from class java.awt.Component**

`BOTTOM_ALIGNMENT, CENTER_ALIGNMENT, LEFT_ALIGNMENT, RIGHT_ALIGNMENT, TOP_ALIGNMENT`

**Fields inherited from interface java.awt.image.ImageObserver**

`ABORT, ALLBITS, ERROR, FRAMEBITS, HEIGHT, PROPERTIES, SOMEBITS, WIDTH`


**Constructor Summary**

`AbstractTrainAndTestSetProducer()`
  
          Creates a new `AbstractTrainAndTestSetProducer` instance.


| **Method Summary** | |
| --- | --- |
| `abstract  void` | `acceptDataSet(DataSetEvent e)`             Subclass must implement |
| `void` | `addTestSetListener(TestSetListener tsl)`             Add a test set listener |
| `void` | `addTrainingSetListener(TrainingSetListener tsl)`             Add a training set listener |
| `boolean` | `connectionAllowed(java.beans.EventSetDescriptor esd)`             Returns true if, at this time, the object will accept a connection according to the supplied EventSetDescriptor |
| `boolean` | `connectionAllowed(java.lang.String eventName)`             Returns true if, at this time, the object will accept a connection according to the supplied event name |
| `void` | `connectionNotification(java.lang.String eventName, java.lang.Object source)`             Notify this object that it has been registered as a listener with a source with respect to the supplied event name |
| `void` | `disconnectionNotification(java.lang.String eventName, java.lang.Object source)`             Notify this object that it has been deregistered as a listener with a source with respect to the supplied event name |
| `BeanVisual` | `getVisual()`             Get the visual for this bean |
| `void` | `removeTestSetListener(TestSetListener tsl)`             Remove a test set listener |
| `void` | `removeTrainingSetListener(TrainingSetListener tsl)`             Remove a training set listener |
| `void` | `setLog(Logger logger)`             Set a log for this bean |
| `void` | `setVisual(BeanVisual newVisual)`             Set the visual for this bean |
| `abstract  void` | `stop()`             Stop any processing that the bean might be doing. |
| `void` | `useDefaultVisual()`             Use the default visual for this bean |

**Methods inherited from class javax.swing.JPanel**

`getAccessibleContext, getUI, getUIClassID, setUI, updateUI`

**Methods inherited from class javax.swing.JComponent**

`addAncestorListener, addNotify, addVetoableChangeListener, computeVisibleRect, contains, createToolTip, disable, enable, firePropertyChange, firePropertyChange, firePropertyChange, getActionForKeyStroke, getActionMap, getAlignmentX, getAlignmentY, getAncestorListeners, getAutoscrolls, getBaseline, getBaselineResizeBehavior, getBorder, getBounds, getClientProperty, getComponentPopupMenu, getConditionForKeyStroke, getDebugGraphicsOptions, getDefaultLocale, getFontMetrics, getGraphics, getHeight, getInheritsPopupMenu, getInputMap, getInputMap, getInputVerifier, getInsets, getInsets, getListeners, getLocation, getMaximumSize, getMinimumSize, getNextFocusableComponent, getPopupLocation, getPreferredSize, getRegisteredKeyStrokes, getRootPane, getSize, getToolTipLocation, getToolTipText, getToolTipText, getTopLevelAncestor, getTransferHandler, getVerifyInputWhenFocusTarget, getVetoableChangeListeners, getVisibleRect, getWidth, getX, getY, grabFocus, isDoubleBuffered, isLightweightComponent, isManagingFocus, isOpaque, isOptimizedDrawingEnabled, isPaintingForPrint, isPaintingTile, isRequestFocusEnabled, isValidateRoot, paint, paintImmediately, paintImmediately, print, printAll, putClientProperty, registerKeyboardAction, registerKeyboardAction, removeAncestorListener, removeNotify, removeVetoableChangeListener, repaint, repaint, requestDefaultFocus, requestFocus, requestFocus, requestFocusInWindow, resetKeyboardActions, reshape, revalidate, scrollRectToVisible, setActionMap, setAlignmentX, setAlignmentY, setAutoscrolls, setBackground, setBorder, setComponentPopupMenu, setDebugGraphicsOptions, setDefaultLocale, setDoubleBuffered, setEnabled, setFocusTraversalKeys, setFont, setForeground, setInheritsPopupMenu, setInputMap, setInputVerifier, setMaximumSize, setMinimumSize, setNextFocusableComponent, setOpaque, setPreferredSize, setRequestFocusEnabled, setToolTipText, setTransferHandler, setVerifyInputWhenFocusTarget, setVisible, unregisterKeyboardAction, update`

**Methods inherited from class java.awt.Container**

`add, add, add, add, add, addContainerListener, addPropertyChangeListener, addPropertyChangeListener, applyComponentOrientation, areFocusTraversalKeysSet, countComponents, deliverEvent, doLayout, findComponentAt, findComponentAt, getComponent, getComponentAt, getComponentAt, getComponentCount, getComponents, getComponentZOrder, getContainerListeners, getFocusTraversalKeys, getFocusTraversalPolicy, getLayout, getMousePosition, insets, invalidate, isAncestorOf, isFocusCycleRoot, isFocusCycleRoot, isFocusTraversalPolicyProvider, isFocusTraversalPolicySet, layout, list, list, locate, minimumSize, paintComponents, preferredSize, printComponents, remove, remove, removeAll, removeContainerListener, setComponentZOrder, setFocusCycleRoot, setFocusTraversalPolicy, setFocusTraversalPolicyProvider, setLayout, transferFocusBackward, transferFocusDownCycle, validate`

**Methods inherited from class java.awt.Component**

`action, add, addComponentListener, addFocusListener, addHierarchyBoundsListener, addHierarchyListener, addInputMethodListener, addKeyListener, addMouseListener, addMouseMotionListener, addMouseWheelListener, bounds, checkImage, checkImage, contains, createImage, createImage, createVolatileImage, createVolatileImage, dispatchEvent, enable, enableInputMethods, firePropertyChange, firePropertyChange, firePropertyChange, firePropertyChange, firePropertyChange, getBackground, getBounds, getColorModel, getComponentListeners, getComponentOrientation, getCursor, getDropTarget, getFocusCycleRootAncestor, getFocusListeners, getFocusTraversalKeysEnabled, getFont, getForeground, getGraphicsConfiguration, getHierarchyBoundsListeners, getHierarchyListeners, getIgnoreRepaint, getInputContext, getInputMethodListeners, getInputMethodRequests, getKeyListeners, getLocale, getLocation, getLocationOnScreen, getMouseListeners, getMouseMotionListeners, getMousePosition, getMouseWheelListeners, getName, getParent, getPeer, getPropertyChangeListeners, getPropertyChangeListeners, getSize, getToolkit, getTreeLock, gotFocus, handleEvent, hasFocus, hide, imageUpdate, inside, isBackgroundSet, isCursorSet, isDisplayable, isEnabled, isFocusable, isFocusOwner, isFocusTraversable, isFontSet, isForegroundSet, isLightweight, isMaximumSizeSet, isMinimumSizeSet, isPreferredSizeSet, isShowing, isValid, isVisible, keyDown, keyUp, list, list, list, location, lostFocus, mouseDown, mouseDrag, mouseEnter, mouseExit, mouseMove, mouseUp, move, nextFocus, paintAll, postEvent, prepareImage, prepareImage, remove, removeComponentListener, removeFocusListener, removeHierarchyBoundsListener, removeHierarchyListener, removeInputMethodListener, removeKeyListener, removeMouseListener, removeMouseMotionListener, removeMouseWheelListener, removePropertyChangeListener, removePropertyChangeListener, repaint, repaint, repaint, resize, resize, setBounds, setBounds, setComponentOrientation, setCursor, setDropTarget, setFocusable, setFocusTraversalKeysEnabled, setIgnoreRepaint, setLocale, setLocation, setLocation, setName, setSize, setSize, show, show, size, toString, transferFocus, transferFocusUpCycle`

**Methods inherited from class java.lang.Object**

`equals, getClass, hashCode, notify, notifyAll, wait, wait, wait`

**Methods inherited from interface weka.gui.beans.BeanCommon**

`getCustomName, isBusy, setCustomName`

**Constructor Detail**

### AbstractTrainAndTestSetProducer

```
public AbstractTrainAndTestSetProducer()
```

:   Creates a new `AbstractTrainAndTestSetProducer` instance.


**Method Detail**

### acceptDataSet

```
public abstract void acceptDataSet(DataSetEvent e)
```

:   Subclass must implement

    :   **Specified by:**: `acceptDataSet` in interface `DataSourceListener`
    :   **Parameters:**: `e` - a `DataSetEvent` value

---


### addTrainingSetListener

```
public void addTrainingSetListener(TrainingSetListener tsl)
```

:   Add a training set listener

    :   **Specified by:**: `addTrainingSetListener` in interface `TrainingSetProducer`
    :   **Parameters:**: `tsl` - a `TrainingSetListener` value

---


### removeTrainingSetListener

```
public void removeTrainingSetListener(TrainingSetListener tsl)
```

:   Remove a training set listener

    :   **Specified by:**: `removeTrainingSetListener` in interface `TrainingSetProducer`
    :   **Parameters:**: `tsl` - a `TrainingSetListener` value

---


### addTestSetListener

```
public void addTestSetListener(TestSetListener tsl)
```

:   Add a test set listener

    :   **Specified by:**: `addTestSetListener` in interface `TestSetProducer`
    :   **Parameters:**: `tsl` - a `TestSetListener` value

---


### removeTestSetListener

```
public void removeTestSetListener(TestSetListener tsl)
```

:   Remove a test set listener

    :   **Specified by:**: `removeTestSetListener` in interface `TestSetProducer`
    :   **Parameters:**: `tsl` - a `TestSetListener` value

---


### setVisual

```
public void setVisual(BeanVisual newVisual)
```

:   Set the visual for this bean

    :   **Specified by:**: `setVisual` in interface `Visible`
    :   **Parameters:**: `newVisual` - a `BeanVisual` value

---


### getVisual

```
public BeanVisual getVisual()
```

:   Get the visual for this bean

    :   **Specified by:**: `getVisual` in interface `Visible`
    :   **Returns:**: a `BeanVisual` value

---


### useDefaultVisual

```
public void useDefaultVisual()
```

:   Use the default visual for this bean

    :   **Specified by:**: `useDefaultVisual` in interface `Visible`

---


### connectionAllowed

```
public boolean connectionAllowed(java.lang.String eventName)
```

:   Returns true if, at this time,
    the object will accept a connection according to the supplied
    event name

    :   **Specified by:**: `connectionAllowed` in interface `BeanCommon`
    :   **Parameters:**: `eventName` - the event **Returns:**: true if the object will accept a connection

---


### connectionAllowed

```
public boolean connectionAllowed(java.beans.EventSetDescriptor esd)
```

:   Returns true if, at this time,
    the object will accept a connection according to the supplied
    EventSetDescriptor

    :   **Specified by:**: `connectionAllowed` in interface `BeanCommon`
    :   **Parameters:**: `esd` - the EventSetDescriptor **Returns:**: true if the object will accept a connection

---


### connectionNotification

```
public void connectionNotification(java.lang.String eventName,
                                   java.lang.Object source)
```

:   Notify this object that it has been registered as a listener with
    a source with respect to the supplied event name

    :   **Specified by:**: `connectionNotification` in interface `BeanCommon`
    :   **Parameters:**: `eventName` - the event: `source` - the source with which this object has been registered as a listener

---


### disconnectionNotification

```
public void disconnectionNotification(java.lang.String eventName,
                                      java.lang.Object source)
```

:   Notify this object that it has been deregistered as a listener with
    a source with respect to the supplied event name

    :   **Specified by:**: `disconnectionNotification` in interface `BeanCommon`
    :   **Parameters:**: `eventName` - the event: `source` - the source with which this object has been registered as a listener

---


### setLog

```
public void setLog(Logger logger)
```

:   Set a log for this bean

    :   **Specified by:**: `setLog` in interface `BeanCommon`
    :   **Parameters:**: `logger` - a `weka.gui.Logger` value

---


### stop

```
public abstract void stop()
```

:   Stop any processing that the bean might be doing.
    Subclass must implement

    :   **Specified by:**: `stop` in interface `BeanCommon`


---


|  |  |  |
| --- | --- | --- |
| **Overview** \| **Package** \| **Class** \| **Tree** \| **Deprecated** \| **Index** \| **Help** \| **Weka's home** | |  |
| **PREV CLASS**   **NEXT CLASS** | **FRAMES**    **NO FRAMES**     **All Classes** |  |
| SUMMARY: NESTED \| FIELD \| CONSTR \| METHOD | DETAIL: FIELD \| CONSTR \| METHOD |  |


---
